# Highly adsorptive removal of cefiderocol during continuous venovenous hemodiafiltration equipped with oXiris filter in an orthotopic liver transplant recipient having septic shock caused by VIM-producing *Klebsiella pneumoniae*

**DOI:** 10.1093/jac/dkaf040

**Published:** 2025-02-17

**Authors:** Milo Gatti, Matteo Rinaldi, Cristiana Laici, Antonio Siniscalchi, Simone Ambretti, Maddalena Giannella, Pierluigi Viale, Federico Pea

**Affiliations:** Department of Medical and Surgical Sciences, Alma Mater Studiorum, University of Bologna, Bologna, Italy; Clinical Pharmacology Unit, Department for Integrated Infectious Risk Management, IRCCS Azienda Ospedaliero-Universitaria di Bologna, Bologna, Italy; Department of Medical and Surgical Sciences, Alma Mater Studiorum, University of Bologna, Bologna, Italy; Infectious Diseases Unit, Department for Integrated Infectious Risk Management, IRCCS Azienda Ospedaliero-Universitaria di Bologna, Bologna, Italy; Division of Anesthesiology, Department of Anesthesia and Intensive Care, IRCCS Azienda Ospedaliero-Universitaria di Bologna, Bologna, Italy; Division of Anesthesiology, Department of Anesthesia and Intensive Care, IRCCS Azienda Ospedaliero-Universitaria di Bologna, Bologna, Italy; Department of Medical and Surgical Sciences, Alma Mater Studiorum, University of Bologna, Bologna, Italy; Department for Integrated Infectious Risk Management, Operative Unit of Microbiology, IRCCS Azienda Ospedaliero-Universitaria di Bologna, Bologna, Italy; Department of Medical and Surgical Sciences, Alma Mater Studiorum, University of Bologna, Bologna, Italy; Infectious Diseases Unit, Department for Integrated Infectious Risk Management, IRCCS Azienda Ospedaliero-Universitaria di Bologna, Bologna, Italy; Department of Medical and Surgical Sciences, Alma Mater Studiorum, University of Bologna, Bologna, Italy; Infectious Diseases Unit, Department for Integrated Infectious Risk Management, IRCCS Azienda Ospedaliero-Universitaria di Bologna, Bologna, Italy; Department of Medical and Surgical Sciences, Alma Mater Studiorum, University of Bologna, Bologna, Italy; Clinical Pharmacology Unit, Department for Integrated Infectious Risk Management, IRCCS Azienda Ospedaliero-Universitaria di Bologna, Bologna, Italy

Continuous renal replacement therapy (CRRT) is a technique useful in critically ill patients for replacing the lost kidney blood-filtering function in the presence of severe renal dysfunction.^1^ In recent years, CRRT has also emerged as a valuable approach for removing the inflammatory cytokine burden in the early phase of septic shock.^2^ In this scenario, equipping CRRT with an oXiris haemofilter in critical septic patients was shown to either enhance inflammatory cytokine adsorption or to reduce organ damage and mortality rate.^2^ Unfortunately, under these circumstances therapeutic drugs may also be consistently removed.

With regard to beta-lactams, current evidence on pharmacokinetic (PK) alterations during CRRT equipped with an oXiris hemofilter is limited.^3^ Here, we describe the PK alterations concerning cefiderocol in a orthotopic liver transplant recipient who, while being treated with continuous infusion (CI) cefiderocol because of having septic shock caused by VIM-producing *Klebsiella pneumoniae* (*Kp*), underwent continuous venovenous hemodiafiltration (CVVHDF) with a traditional AN-69 ST150 filter initially, and subsequently with a highly adsorptive oXiris filter.

A 51-year-old male underwent orthotopic liver transplant because of complicated alcoholic cirrhosis (Model for End-Stage Liver Disease score of 20). The post-operative course was complicated by acute kidney injury requiring CVVHDF, rectal colonization by VIM-producing *Kp* and liver failure caused by post-traumatic thrombosis of the suprahepatic vein. The patient needed liver re-transplantation on day 7, and on days 11–27 and 35–44 suffered from bloodstream infection and ventilator-associated pneumonia (VAP) caused by VIM-producing *Kp*. On these occasions, treatment courses with CI ceftazidime-avibactam plus CI aztreonam were given. Unfortunately, on day 45, the patient needed a second re-transplantation because of graft rejection. On day 52, clinical conditions suddenly worsened, and septic shock as a complication of VIM-producing *Kp*-related VAP occurred (>10^6^ cfu/mL). Being a clinical isolate susceptible to cefiderocol (tested by disc diffusion as suggested by EUCAST and interpreted according to clinical breakpoint, namely MIC ≤2 mg/L),^4^ treatment with a 2 g loading dose of cefiderocol followed by a 2 g q8h over an 8-h (CI) maintenance dose was started, according to previous findings concerning cefiderocol PK behaviour during CVVHDF.^5^ Cefiderocol exposure was optimized by means of a real-time therapeutic drug monitoring (TDM)-guided approach with the intent of maximizing aggressive pharmacokinetic/pharmacodynamic (PK/PD) target attainment against all the susceptible pathogens. The desired target was a free steady-state concentration (*fC*_ss_) of >8 mg/L, corresponding to a *fC*_ss_-to-MIC ratio >4 against pathogens with an MIC value up to the clinical breakpoint of 2 mg/L.^5^ For this purpose, cefiderocol total plasma *C*_ss_ values were measured by means of a validated liquid chromatography with tandem mass spectrometry method,^6^ and the free moiety (*fC*_ss_) values were calculated by considering 42% of the total *C*_ss_, based on a plasma protein binding rate of 58%.^7^ Cefiderocol plasma *C*_ss_ values were measured in real time on days 53, 55, 60 and 63, and prompt TDM-guided advice for cefiderocol dosing on each occasion was provided.^8^ At each TDM assessment, cefiderocol total clearance (CL_tot_) was calculated by means of the following formula: CL_tot_ (L/h) = infusion rate (mg/h)/*C*_ss_ (mg/L). Data on cefiderocol PK features, PK/PD target attainment and CVVHDF operative conditions are summarized in Figure 1. Specifically, CVVHDF was performed by means of a Prisma Flex System equipped with an AN69 high-flux ST-150 filter membrane and using citrate as a regional anticoagulant. Blood flow rate, pre-blood pump flow rate, post-filter replacement fluid rate and dialysate flow rate were set in the ranges of 110–150, 917–1250, 400–500 and 400–700 mL/h, so that the delivered CVVHDF dose was in the range of 30–35 mL/kg/h. The desired PK/PD target was always attained. Unfortunately, after an initial clinical improvement, on day 60 the patient experienced a further episode of septic shock requiring vasopressor support and mechanical ventilation. The oXiris filter was then adopted for further increasing the removal of the inflammatory cytokine burden. It is worth noting that, although in this latter phase the CVVHDF total effluent flow rate (*Q*_ef_) decreased by ∼10%, the cefiderocol CL_tot_ under the oXiris filter increased by 49.6% compared to that under the AN-69 ST150 filter (4.33 versus 2.90 L/h). Unfortunately, bronchoalveolar lavage performed on day 65 yielded both VIM-producing *Kp* resistant to cefiderocol and *Burkholderia cepacia*, so the treatment was switched to combination therapy with imipenem-relebactam plus aztreonam.

**Figure 1.**
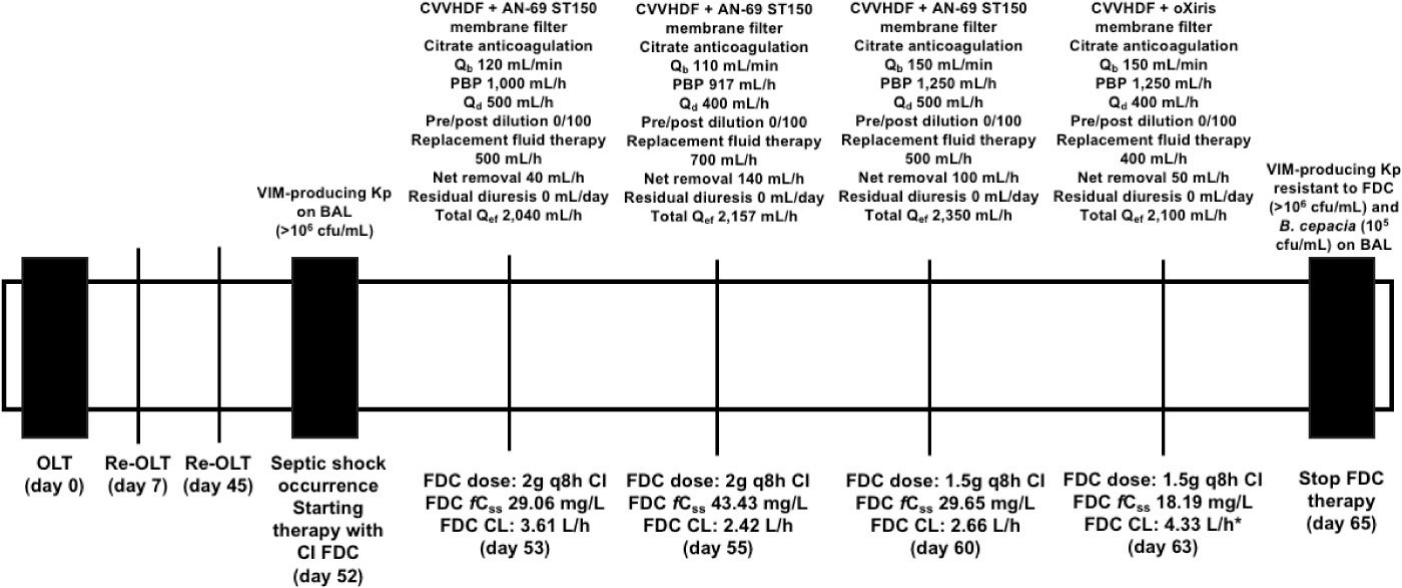
Cefiderocol PK features and CVVHDF operative conditions at each TDM assessment. BAL, bronchoalveolar lavage; CL, clearance; FDC, cefiderocol; OLT, orthotopic liver transplantation; PBP, pre-blood pump flow rate; *Q*_b_, blood flow rate; *Q*_d_, dialysate flow rate; *Q*_ef_, total effluent flow rate. * percentage variation in FDC CL between average CL during AN-69 ST150 (2.90 L/h) and oXiris filter membrane (4.33 L/h) equal to 49.6%, and calculated according to the following equation: [(FDC CL during oXiris − average FDC CL during AN-69 ST150)/(average FDC CL during AN-69 ST150)] × 100%.

To the best of our knowledge, this is the first case reporting cefiderocol PK features during CVVHDF equipped with an oXiris membrane filter. Notably, equipping CVVHDF with this filter membrane resulted in a consistent increase of cefiderocol removal compared to the traditional AN-69 ST150 filter membrane. Overall, considering that the CVVHDF dose under oXiris treatment was partially decreased compared with that under AN-69 ST150 treatment, we are confident that the net effect on decreasing *C*_ss_ under oXiris treatment could probably be mainly attributed to an adsorptive removal by the oXiris membrane filter. Notably, it could be speculated that the multilayer linear structure cationic complex resulting from the polyethyleneimine and heparin coatings of the oXiris membrane filter might have caused relevant adsorption of cefiderocol, this being a negatively charged molecule,^9^ similar to what was previously shown for other negatively charged molecules.^2^ Our findings are in agreement with a previous population PK study conducted among 12 critically ill patients showing that the meropenem CL_tot_ during CVVHDF equipped with an oXiris filter was higher than that reported during CVVHDF equipped with an AN-69 filter.^3^

Consequently, clinicians should be aware that, during cefiderocol treatment, a potential dosage increase could be needed for granting optimal exposure when shifting CVVHDF equipment from the traditional AN-69 ST150 filter to the highly adsorptive oXiris filter. In these cases, adopting a real-time TDM-guided strategy may be helpful for promptly implementing dosing adaptation in relation to the evolving pathophysiological and/or iatrogenic conditions to attain cefiderocol aggressive PK/PD targets. Further larger prospective studies are warranted for testing our hypothesis.

## Transparency declarations

M.G. reports grant from Angelini, and participated in advisory board for AdvanzPharma and Viatris outside the submitted work. P.V. has served as a consultant for bioMérieux, Gilead, Merck Sharp & Dohme, Nabriva, Nordic Pharma, Pfizer, Thermo-Fisher and Venatorx, and received payment for serving on the speaker’s bureaus for Correvio, Gilead, Merck Sharp & Dohme, Nordic Pharma and Pfizer outside the submitted work. F.P participated in speaker bureau for Angelini, BeiGene, Gilead, InfectoPharm, Menarini, Merck Sharp & Dohme, Pfizer and Shionogi, and in advisory board for BeiGene, Merck Sharp & Dohme, Pfizer and Viatris outside the submitted work. The other authors report no potential conflicts of interest for this work.
